# On the generation of realistic synthetic petrographic datasets using a style-based GAN

**DOI:** 10.1038/s41598-022-16034-4

**Published:** 2022-07-27

**Authors:** Ivan Ferreira, Luis Ochoa, Ardiansyah Koeshidayatullah

**Affiliations:** 1 Department of Geosciences, College of Petroleum and Minerals, King Fahd University of Petroleum and Minerals, Dhahran, Saudi Arabia; 2 Departamento de Geociencias, Universidad Nacional de Colombia, Bogotá, Colombia

**Keywords:** Geology, Mineralogy, Petrology, Sedimentology, Computer science

## Abstract

Deep learning architectures have transformed data analytics in geosciences, complementing traditional approaches to geological problems. Although deep learning applications in geosciences show encouraging signs, their potential remains untapped due to limited data availability and the required in-depth knowledge to provide a high-quality labeled dataset. We approached these issues by developing a novel style-based deep generative adversarial network (GAN) model, *PetroGAN*, to create the first realistic synthetic petrographic datasets across different rock types. *PetroGAN* adopts the architecture of StyleGAN2 with adaptive discriminator augmentation (ADA) to allow robust replication of statistical and esthetical characteristics and improve the internal variance of petrographic data. In this study, the training dataset consists of > 10,000 thin section images both under plane- and cross-polarized lights. Here, using our proposed novel approach, the model reached a state-of-the-art Fréchet Inception Distance (FID) score of 12.49 for petrographic images. We further observed that the FID values vary with lithology type and image resolution. The generated images were validated through a survey where the participants have various backgrounds and level of expertise in geosciences. The survey established that even a subject matter expert observed the generated images were indistinguishable from real images. This study highlights that GANs are a powerful method for generating realistic synthetic data in geosciences. Moreover, they are a future tool for image self-labeling, reducing the effort in producing big, high-quality labeled geoscience datasets. Furthermore, our study shows that PetroGAN can be applied to other geoscience datasets, opening new research horizons in the application of deep learning to various fields in geosciences, particularly with the presence of limited datasets.

## Introduction

Advances in artificial intelligence and machine learning in the last decades have accelerated the process of digital transformation in geosciences and helped to generate meaningful insights from geological data like never before, using a vast array of algorithms^1–4^. Recently, with the advent of generative models like Generative adversarial networks (GANs)^5^, Variational Auto-Encoders (VAEs)^6^, transformer GANs^7^, and Diffusion models^8^, applications of deep learning have led to state-of-the-art results in various aspects, including geosciences. In addition, some reports reveal that the outcomes of these generative models could match geologist-level analysis in various aspects of visual recognition (Table 1). Studies have demonstrated that GANs are a powerful tool to generate realistic and diverse images in an unsupervised manner and are already adopted in several fields, including superresolution, image-to-image translation, text-to-image translation, style-mixing, and generation of realistic images (Table 1). In general, GANs objective is to capture the data distribution via a minimax two-player game that aims to produce synthetic samples based on the original dataset, mimicking its statistical and esthetical characteristics^5^ even going as far as deceiving human observers in the ability to discriminate real images from generated ones^9–11^. In recent years, geosciences have adopted deep learning-based analytics in their workflows, such as image processing tasks. However, the lack of high-quality labeled, varied, and sufficiently large datasets^12^ has resulted in images being overtrained and overfit to certain geological contexts^13^, or there is insufficient data to yield satisfactory results with deep learning algorithms such as Convolutional Neural Networks (CNNs)^14^. As a result, transfer learning has been suggested as an alternative approach^4,15,16^ to avoid or minimize the risk of overtraining in a single geological context using such an approach. Furthermore, the high accuracy obtained from the transfer learning methods creates another dimension of uncertainty whereby a model trained to recognize animals or other daily objects can be applied to classify geological images, such as seismic and petrographic images that have entirely different distributions and features (high- and low-level). Therefore, there is still a gap in image processing and analysis that generative models could help address by bringing deep learning applications closer to geological tasks.

**Table 1:** Applications of GANs for geological data generation.

References	GAN Algorithm	Problem Addressed
^2^	Volumetric DCGAN	Reconstruction of porous media from images of sedimentary rocks
^28^	Dimension Augmenter GAN	Generate 3D Stochastic fields from 2D images for hydrology
^31^	Conditional GAN	Reconstruction and classification of carbonate thin sections
^29^	Cycle-in-Cycle GAN	Improve the resolution of 3D micro-CT images
^27^	Progressive growth GAN	Generation of 2D geological facies models
^30^	GAN and Ensemble Kalman filter	Assisted history matching for a Deepwater lobe system
^32^	SRGAN and ESRGAN	Image Superresolution of Micro-CT images
^33^	DCGAN, LSGAN and WGAN	Time-series generation of mud logs

Historically, clustering techniques and edge detection algorithms were the main algorithms for digital image processing^17^. Recent studies have explored and utilized deep CNNs to address visual recognition and image processing in geosciences^4,17^. The implementation of GANs in geosciences, particularly the petrographic image dataset, has been limited and still relatively under-explored. One of the significant limitations to applying GANs in petrographic images is that most GAN architectures require a massive amount of data, as illustrated in the first implementation of GAN with the MNIST and CIFAR datasets (60,000 images each)^5^. Additionally, the use of image datasets in the 1000s range has led to the overfitting of the generator in GANs^18^. This work addresses the implementation of GANs for petrographic images by (i) experimenting with different dataset sizes, (ii) using various image resolutions, and (iii) applying truncation values to create a framework to generate realistic synthetic petrographic datasets. Our work uses the adaptive discriminator augmentation (ADA) application of StyleGAN2^19–21^ to realistically generate synthetic petrographic images due to the high-quality of generated images by this model, backward compatibility, stability, and the viability of its use in nonfacial generation datasets. In addition, employing the state-of-the-art Fréchet Inception Distance (FID)^22^ scores provides a better metric to evaluate the generated images. The dataset size limitation in petrographic thin sections is a problem addressed in this paper, as images collected and sliced were of a sufficient volume after preprocessing to produce meaningful results using this generative model. The ultimate objectives of this work are to

1. Explore the best image resolution and dataset size to generate realistic thin sections.
2. Develop a novel deep learning framework to generate petrographic synthetic datasets.
3. Discuss the properties of a petrographic GAN model using latent space, transfer learning, interpolation, truncation, and feature extraction^21,23–25^.
4. Evaluate the synthetic datasets through a simple survey from subject matter experts.

We further aim to highlight the application of GAN algorithms and other generative models as a way forward for exploring self-labeling and image generation tasks and how it could support the successful execution of deep learning algorithms and provide a novel workflow for image analysis in geosciences.

### Related work

Recently, GANs have been widely adopted in geosciences with the motivation to explore and apply generative models to generate and manipulate a latent space associated with the geological data of interest, i.e., the highdimensional space where a representation of the data is encoded^2,26,27^. This space is used to upscale the dimensionality and upsample the quality of image datasets. Previous works have demonstrated the far-reaching impact and application of GANs in geosciences, from reservoir simulation to history matching (see Table 1)^2,27–30^.

Furthermore, GANs have been proposed as a tool to create synthetic carbonate components^4^ and for obtaining superresolution micro-computed tomography (Micro-CT) images^29,32^ for digital rock physics workflows. Additionally, GANs have also been used successfully to assist in the reconstruction and classification of carbonate thin sections^31^, positioning GANs as a possible tool to enhance carbonate lithology interpretation workflows in combination with core images and Fullbore Formation MicroImager (FMI) images. Recent applications have also repurposed GANs designed for 2D image generation to 1D time-series generation, an implementation that could have extensive applications in the geosciences^33^.

## Methods

In this study, the datasets consisted of cross-polarized (XPL) and plane-polarized (PPL) RGB thin section images. Information from XPL and PPL images is crucial to determine the type of minerals and lithological variations in thin sections. The datasets were prepared using (i) the provided dataset tool generation from the StyleGAN repository^20,21^ and (ii) image slicing as a data augmentation technique. The StyleGAN architecture was selected based on its state-of-the-art (SoTA) scores and the ability to experiment with the generated latent space (Table 2),^20,21^. This architecture and its derivatives are the current SoTA for unconditional image generation with the CIFAR-10 dataset (Table 3)^34^.

**Table 2:** Comparison of Fréchet Inception Distance (FID) scores of images generated at 256 × 256 for different dataset sizes from the Flickr Faces HQ Dataset (FFHQ) using StyleGAN2 with BigGAN, and the StyleGAN2 + ADA. Adapted from the StyleGAN2 + ADA original paper^21^.

GAN Implementation	2k Training set	5k Training set	10k Training set	140k Training set
BigGAN^23^	60.47	32.34	15.85	11.08
StyleGAN2^20^	66.77	39.42	8.80	3.81
+ ADA21,35	15.76	10.78	5.40	3.79

**Table 3:** CIFAR-10 FID score benchmark for unconditional image generation. GAN: Generative Adversarial Network; VAE: Variational AutoEncoder. Diffusion: Diffusion model. StyleGAN and derivatives in boldface.

References	Model	↓ FID	Type	Year
^36^	**StyleGAN-XL**	1.85	GAN	2022
^37^	LSGM	2.10	VAE	2021
^38^	Subspace Diffusion	2.10	Diffusion	2021
^39^	CLD-SGM	2.23	Diffusion	2021
^40^	**StyleGAN2 + DiffAugment + D2D-CE**	2.24	GAN	2021
^41^	INDM	2.28	Diffusion	2021
^40^	**StyleGAN2 + ADA**	2.32	GAN	2021
^40^	**StyleGAN2 + ADA + D2D-CE**	2.32	GAN	2021
^41^	UDM	2.33	Diffusion	2021
^42^	BDDM	2.38	Diffusion	2022

### PetroGAN architecture

**Figure 1:**
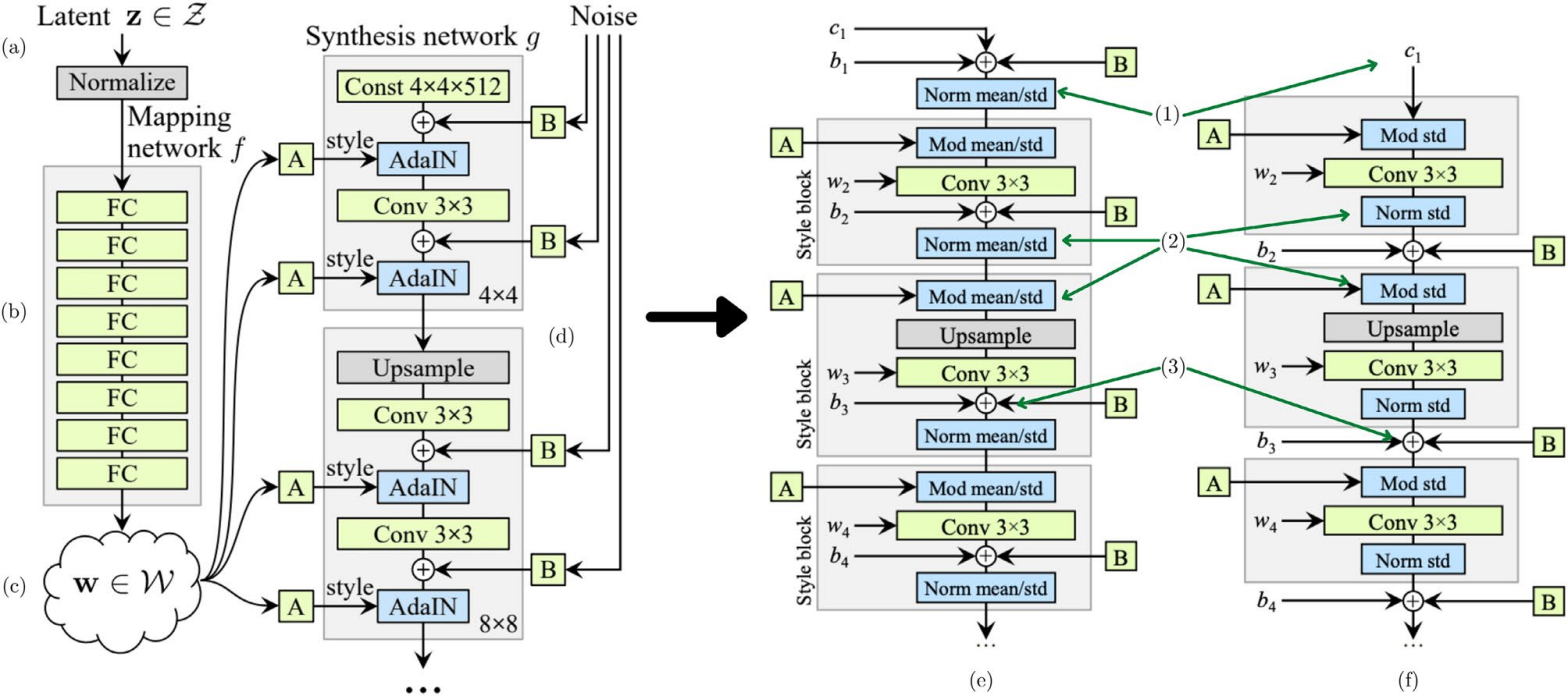
Original StyleGAN architecture, (**a**) The latent vector z introduced, (**b**) eight fully connected layers used to obtain, (**c**) latent code w containing the features and (**d**) a series of AdaIN, normalization and convolutions using progressive growth to generate high- resolution images^19^. Major modifications were made to (**e**) StyleGAN in (**f**) StyleGAN2. The model is simplified by removing the initial processing of the constant (1), the removal ofremoving the mean in the process of normalizing the features (2), and the transfer of the noise module (+) outside of the style block (3). M(modified from the StyleGAN and StyleGAN2 papers)^19,20^.

In this work, the proposed GAN model, *PetroGAN*, adopts a style-based GAN architecture (Fig. 1). The model consists of (i) a mapping network from the latent vector z (i.e., the latent vector representation of an image in latent space Z), (ii) a mapping of this vector using eight fully connected layers in the W Space the space of all style vectors w, and (iii) used in conjunction with Adaptive Instance Normalization (AdaIN) layers^35^ to control the features in the generator. This is managed using progressive image growth, reducing the complexity of generating high-resolution images by taking a step-by-step approach^19^. However, this has been linked to the production of artifacts in the generated image, one of the main reasons behind the re-engineering of the model adopted in StyleGAN2^20,21^.

Eq. (1) is a special normalization operation where the input feature map, *x*_*i*_, is normalized by instance, then scaled and biased using the style information, *µ* being the mean and *σ* the standard deviation of *x*_*i*_, with *y*_*s,i*_ and *y*_*b,i*_ being a pair of style values^21^.

1 $$\mathrm{AdaIN}\left({\mathbf{x}}_{i},\mathbf{y}\right)={\mathbf{y}}_{s,i}{\mathbf{x}}_{i}-\mu \left({\mathbf{x}}_{i}\right)/\sigma \left({\mathbf{x}}_{i}\right)+{\mathbf{y}}_{b,i}$$

For this reason, we use the second iteration of the StyleGAN line of models, Fig. 1f^20,21^, which further developed the original StyleGAN^19^. This architecture is constantly developed and improved and has backward compatibility with the preceding StyleGAN architectures with the same dataset preparation tools, accepted image resolutions, and workflows utilized. Although the latest iteration of this model is StyleGAN3^43^, we did not choose this architecture because an acceptable FID score was not achieved, and the model diverged with the same dataset size. The generation of unintended artifacts in StyleGAN, primarily due to the progressive growth technique, was addressed by creating the StyleGAN2 model^20,21^. This was achieved by simplifying and eliminating steps in the architecture (Fig. 1f). Instead of using progressive growth to generate high-resolution images, we employed skip connections in StyleGAN2^20^. This method allows skipping some layers in the model and feeding this output to the subsequent layers as realized in the Residual Networks (ResNet) architectures^44^. Style-mixing is a different application of this architecture, with styles extracted after the fully connected layers by an Affine transform (A in Fig. 1e); these style blocks further extract coarser and fine styles from an image dataset. For a facial dataset, this ranges from pose (coarse) to eye color (fine). The style blocks of this architecture consist of modulation, convolution, and normalization layers; the style block starts with a modulation operation, Eq. (2), being applied, which scales each input feature from the extracted style^21^.

2 $${w}_{ijk}^{{{\prime}}}={s}_{i}\cdot {w}_{ijk}$$

where *w* and *w*′ are the modulated weights and *s*_*i*_ is the scale for each input. This is followed by a 3 × 3 convolution operation, finalizing the style block with a normalization of the weights using Eq. (3), with a constant *ε* added to avoid instability during training.

3 $${w}_{ijk}^{{{\prime}}{{\prime}}}={w}_{ijk}^{{{\prime}}}/\sqrt{\sum_{i,k} {w}_{ijk}^{{{\prime}}}{{\prime}}+\varepsilon }$$

### Dataset sources

**Figure 2:**
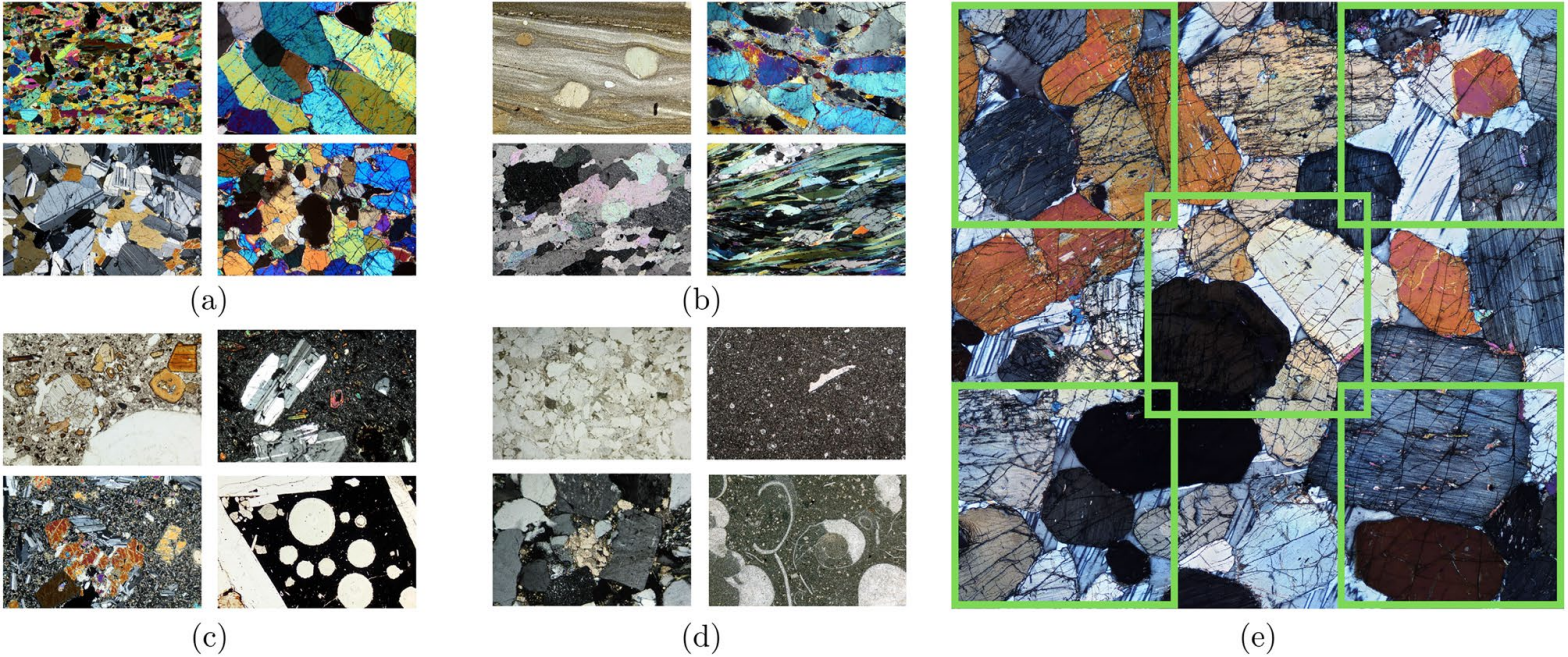
Four lithology classes extracted from Streckeisen dataset: (**a**) plutonic, (**b**) metamorphic, (**c**) volcanic and (**d**) sedimentary rocks in thin sections, (**e**) example of image slicing applied to the VPM dataset. Image slicing was applied to MacQuarie university images, splitting the original into five representative subsections.

Petrographic images were collected from publicly available sources, as listed in Table 4. The dataset consists of high-resolution images of 1701 × 1686 pixels collected from the Virtual Petrographic Microscope project (VPM) in PPL and XPL with different rotation angles for each image^45^ (Fig. 2e). This dataset is complemented by 800 × 533 pixel petrographic images taken from the Strekeisen project (Fig. 2a–d)^46^ Images from all datasets were divided into four main rock types: (1) plutonic, (2) volcanic, (3) metamorphic, and (4) sedimentary classes. Magnifications were also considered to obtain several representations of various minerals, ranging from 10× and 20× from the Strekeisen project and the Atlas of sedimentary rocks book^47^, 4× from JD. Derochette project^48^, and full thin section photomicrographs for the VPM.

### Image slicing and final dataset

Data processing was performed through standard image manipulation made available as part of the StyleGAN2 application, Numpy^49^, OpenCV, Pillow, and PyTorch^50^. As per the requirements of the StyleGAN2 architecture, images needed to be in a square format with dimensions in powers of two (i.e., 32×32, 256×256, 512×512 px, etc.). The original images were cropped or sliced to 512x512 px to achieve a sizable dataset for the GAN to train and satisfy the StyleGAN2 dataset requirement^18–20^ using the highest possible resolution while preserving the essential features of the petrographic dataset. The final dataset consisted of 10070 petrographic images belonging to four classes; this combined set of images is used to train the GAN for generating 512x512 px images. One of the main objectives of the generated dataset was to achieve greater than 10k images and have a class balance between lithologies, as shown in Table 5.

**Table 4:** Data precedence for thin sections used for training, SP: Strekeisen Project, VPM: Virtual Microscope Project, Adams: Atlas of sedimentary rocks [Adams et al., 1984], JD: J. M. Derochette.

Source	Number of images (Quantity per dataset)	Average resolution	Class	Images sliced (512 × 512)
SP, VPM	(2549,20)	800 × 533, 1701 × 1686	Igneous plutonic	2645
SP, VPM, JD	(1756,60,45)	800 × 533, 1701 × 1686, 2600 × 1700	Igneous volcanic	2281
SP, VPM, Adams	(695,150,217)	800 × 533,1701 × 1686, 1120 × 820	Sedimentary	2530
SP, VPM	(2086,132)	800 × 533,1701 × 1686	Metamorphic	2614

### Training procedures

#### Stage I

As a Minimum Viable Product (MVP), training was conducted using only igneous images consisting of 15,294 images with 32×32 pixels in size. Images were taken exclusively from the igneous rocks available from the VPM and SP. The objective of this test was to ensure that convergence in the model was viable, as training time for GAN models usually needs extensive training and high-end computing capabilities entailing one or several Graphical Processing Units (GPU). The MVP trained for four days and 13 hours, using a Quadro M4000 with 8 Gb of video RAM, 30 Gb of RAM, and an eight-core CPU; the model converged and achieved an FID score of 7.49.

#### Stage II

The images were set to a standard size of 512x512 px, which was the maximum size possible with the available dataset. The final dataset consisted of 10,070 representative images of thin sections in both XPL and PPL from four different classes; (i) plutonic, (ii) volcanic, (iii) metamorphic, and (iv) sedimentary rocks. Moreover, the initial model was evaluated using the FID score when it reached 80 Kimgs to assess the training speed. The following model was evaluated every 140 Kimgs processed. Additional models were trained using 256x256 and 128x128 px versions of the dataset with to evaluate how well the FID score performed under various resolutions while keeping the same dataset size. The training was terminated when the values did not improve and started oscillating, i.e., convergence, the model with the lowest FID score was selected. The training was conducted using a Quadro RTX 5000 with 16 Gb of video RAM, 30 Gb of RAM, and an eight-core GPU taking (1) 264 GPU hours for the 512x512 px model, (2) three days and five hours for the 256x256 px model, and (3) 72 GPU hours for the 128x128 px model.

#### Stage III

To evaluate the model’s capability to adapt to specific lithologies, we tested the 512 × 512 model as a starting step for generating domain-specific thin section models. With this in mind, we used three lithology classes from the original dataset, using data augmentation and slicing on the original dataset. The main goal is to generate the highest number of domain-specific lithologies without the limitation of class balancing, previously used in the all-lithology model. The training was resumed using the 512 × 512 all-lithology model and trained during 1120 Kimgs, and it lasted for 44 h with the GPU used.

### Metrics

**Figure 3:**
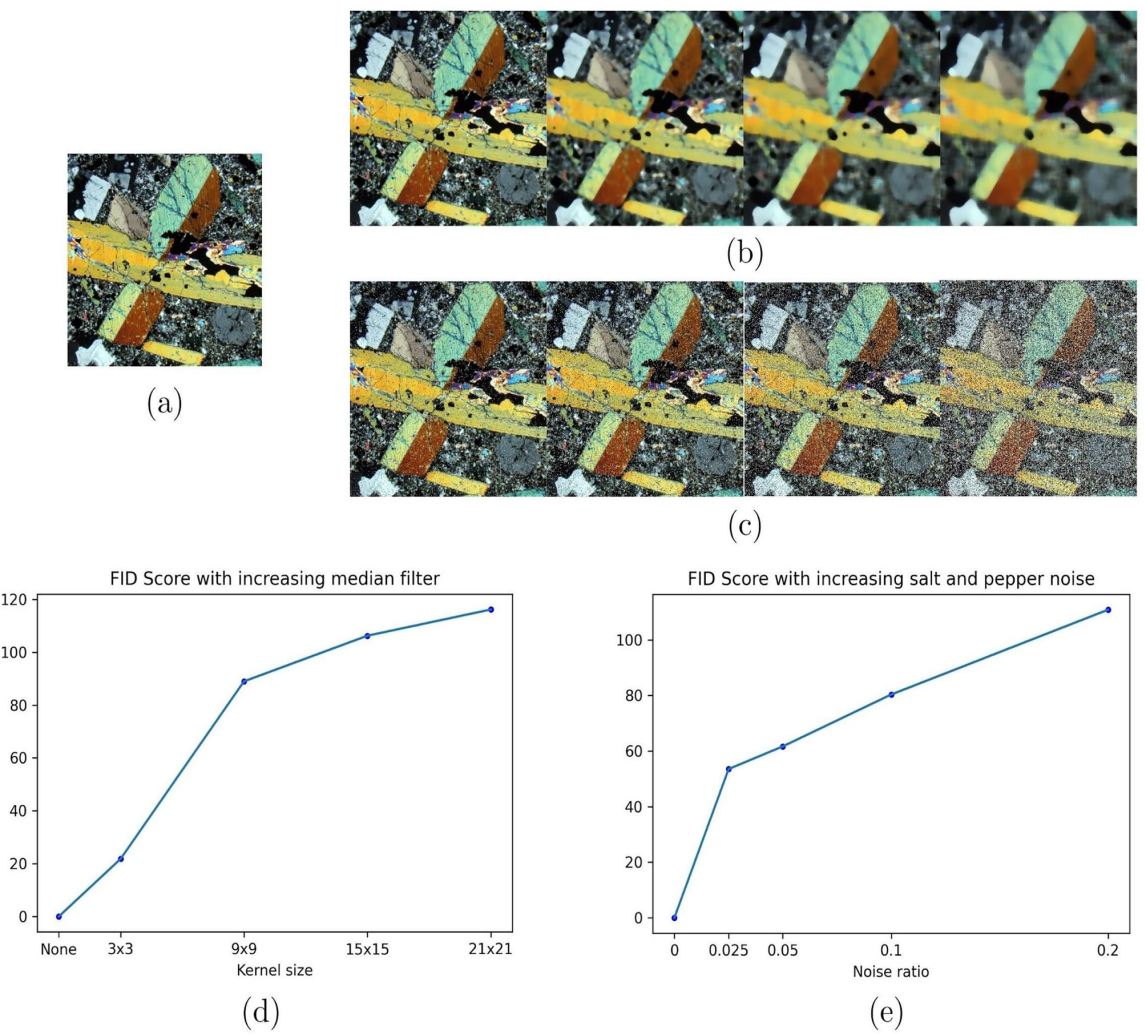
Comparison of different disturbances on FID score for a nepheline foidite from Streckeisen dataset; (**a**) Original image, (**b**) increasing the kernel size median filter applied to the original image, (**c**) adding salt and pepper noise to the image, (**d**) increase in FID score for corresponding kernel size in median filter, and (**e**) increasing the noise-to-signal ratio effect on FID score.

Several metrics help evaluate a GAN performance, such as the FID score, Inception score, and evaluation with domain experts. The most used and state-of-of-the-art metric is the Fréchet Inception Distance score^9,19,21,22^, which is a way of capturing the similarity of generated images to real ones; it is better than the other metrics like the Inception score^24^. In addition, this metric evaluates the statistical distribution of the generated images and its proximity to the statistical distribution of real images, using the last layer of the InceptionV3 model to capture features of the generated and real images, summarizing the activation as a multivariate Gaussian distribution, and calculating its means and covariance^22^. Finally, the distance between the distributions, real and fake, is computed using the similarity via the Fréchet distance^22^. Figure 3 illustrates the behavior of the FID scores reacting to progressive image contamination in the context of petrographic images. The lower the FID score, the closer two image distributions, i.e., the closer a generated image dataset is to real images.

### Visual evaluation

**Figure 4:**
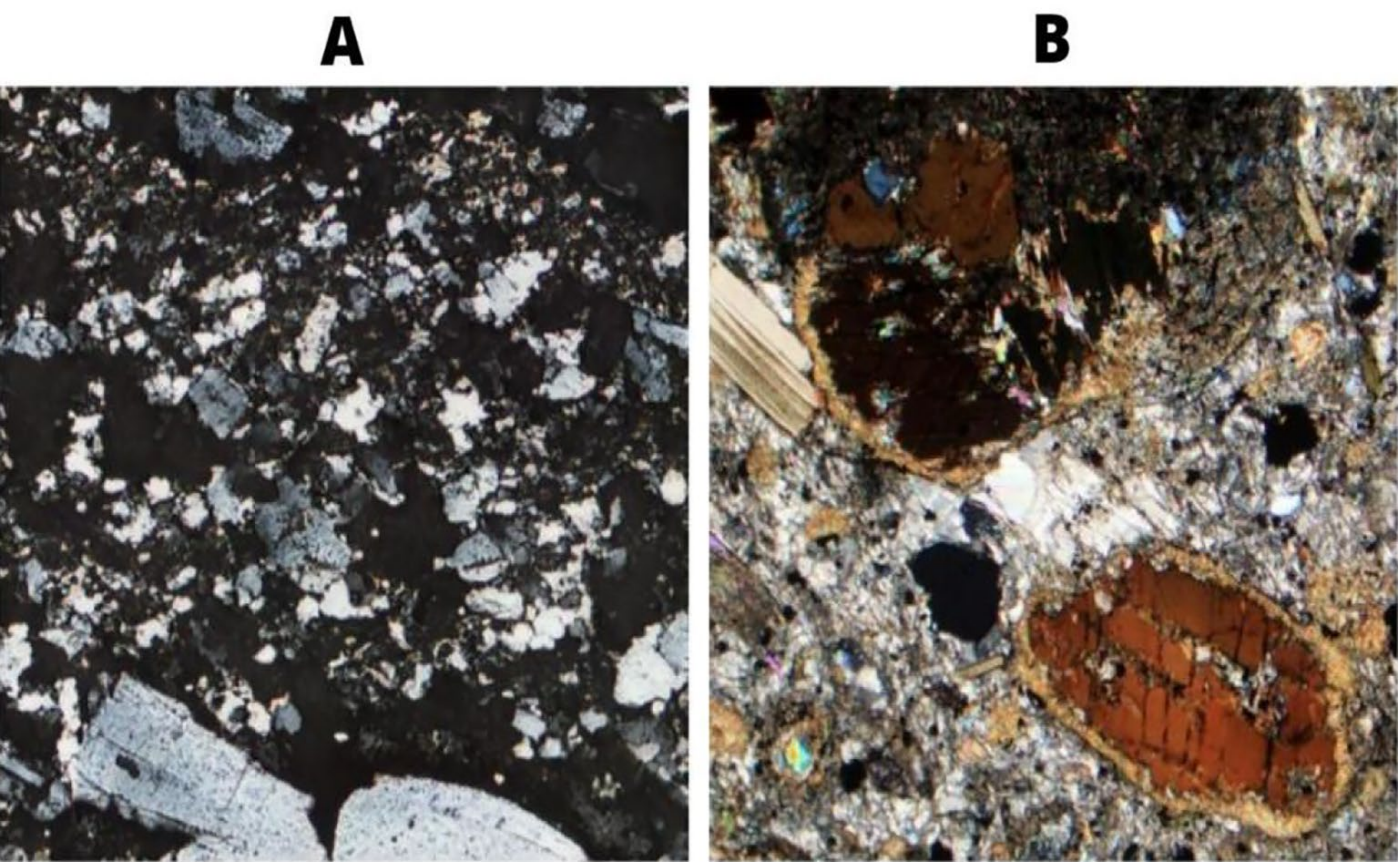
First question presented on the survey. Two images side by side with three answers options, the right image (**B**) being the real one in this case.

As an additional step for evaluating the performance of the final model, a survey was made to assess if the generated thin sections were indistinguishable from the real ones; this survey was aimed at subject matter experts from academia and industry with both geoscience and non-geoscience backgrounds, globally. In the survey, ten actual petrographic images were selected randomly from the training dataset, and ten randomly generated artificial images with randomly selected seed numbers were compared. In addition, the location of the correct image was also randomized. For example, the correct image is on the right and corresponds to an Aillikite from the Strekeisen dataset, and the generated image to the left corresponds to seed 0008 in the model, as seen in Fig. 4. In total, more than two hundred responses were received during a short three-day survey.

## Results

### Model performance

**Figure 5:**
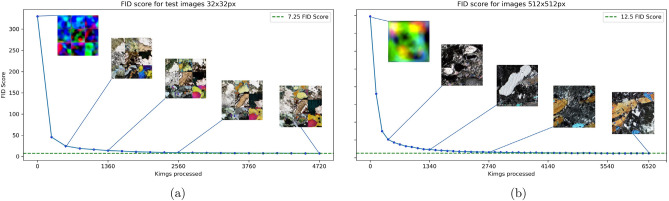
Fréchet Inception Score evolution for (**a**) 32x32 training converging around FID score of 7.24. A 3 × 3 grid of image evolution during training, and (**b**) evolution of FID Score for the 512x512 primary training converges around FID score of 12.5.

The FID score obtained for the reduced size 32x32 px model was low compared with other resolutions, and the final FID score obtained was 7.5 for this dataset (Fig. 5a). A timelapse of the generated images for the 32x32 and 512x512 px models is shown in (Fig. 5). The images reveal the evolution of a 3 × 3 grid of images from noise to low-resolution artificial thin sections in the 32x32 px model and a single thin section in the 512x512 px model. For every 240 Kimgs processed, the FID score was evaluated for the 32x32 px pixel dataset. Furthermore, it was evaluated for the 512x512 px pixel image dataset for every 140 Kimgs. As stated in the methods section and after proving that a generative model using StyleGAN2 was feasible with the MVP, the network was trained with 512x512 px resolution images, and the FID score obtained was 12.49 (Fig. 5b). Based on the literature review, this is encouraging because this is a state-of-the-art FID score for a GAN model trained on microphotographs encompassing all three lithologies. In training, the FID score stabilized at around 2740 Kimgs, and no significant increase was observed after 6520 Kimgs; hence, we obtained the lowest FID score achieved as the final model.

The final model was used to train specific petrographic groups of thin sections of various dataset sizes. Using it as a way of transfer learning and style-mixing in the context of GANs, training was stopped at 1120 Kimgs for each lithology compared to the 6520 Kimgs reached by the original model. Different lithologies and image sizes were trained. A summary of training iterations is provided in Table 5.

**Table 5:** Value of FID scores obtained on different lithologies and dataset sizes. Models in boldface were trained from scratch *models trained using transfer learning applied to all lithologies 512x512 model.

Lithology	Resolution	Dataset size	Training Kimgs	FID score	GPU used
All lithologies	512 × 512	10,070	6520	12.49	RTX5000
Sedimentary*	512 × 512	5995	1120	24.19	RTX5000
Metamorphic*	512 × 512	13,325	1120	14.40	RTX5000
Igneous*	512 × 512	11,350	1120	16.11	RTX5000
All lithologies	256 × 256	10,070	6160	11.89	RTX5000
All lithologies	128 × 128	10,070	8320	10.41	RTX5000
Igneous plutonic	32 × 32	15,294	4720	7.24	M4000

### Synthetic petrographic images

**Figure 6:**
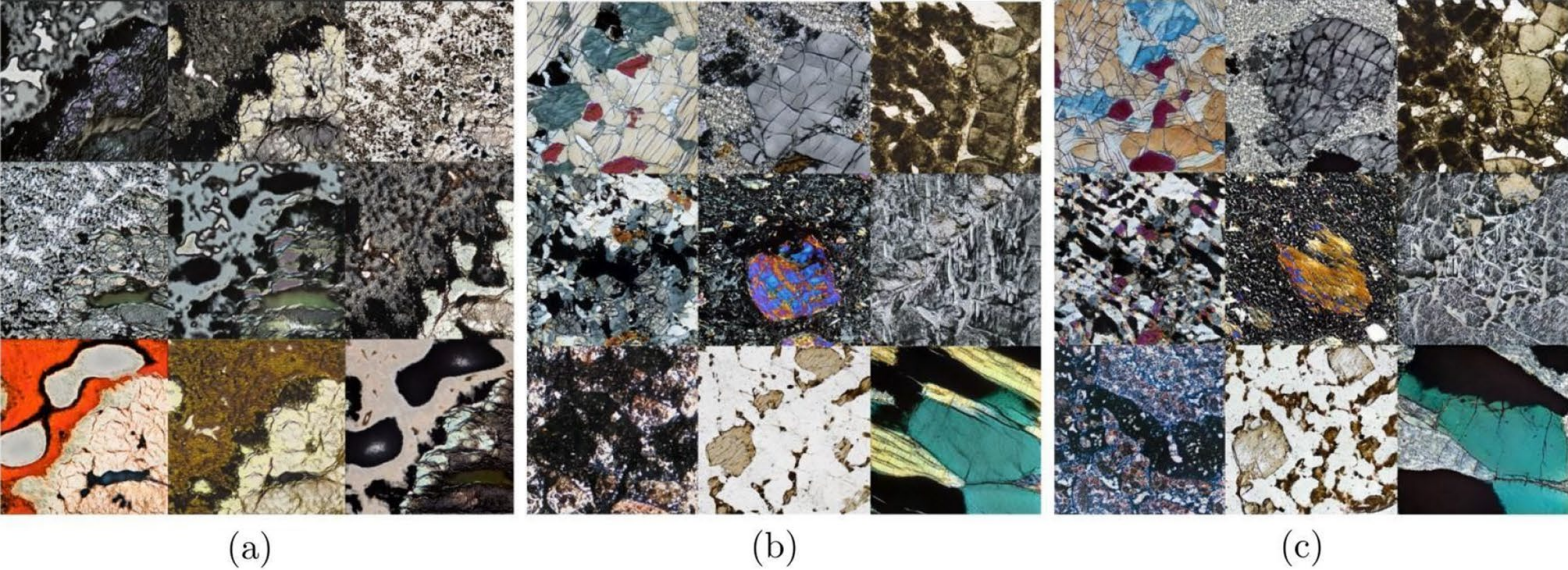
Comparison of different stages of training for selected images that generated (**a**) 74.59, (**b**) 32.84, and (**c**) 12.49.

The images were generated in grids when the FID score was calculated every 140 Kimgs in the case of the 512 × 512 px model, evaluating the progressive improvement in the quality of the generated images, as shown in Fig. 5. The GAN starts from random noise and progressively improves until it reaches convergence, i.e., the point where no further training would improve the model, as seen in Fig. 5. The grid visualization also helps spot mode collapse, whereby the generator becomes proficient at producing one thin section and only generates variants of that image. Nine selected generated images are shown in Fig. 6 with different FID scores during the training of the 512 × 512 model; the seeds were the same, showing a progressive improvement of mineral-like structures in the synthetic images.

### Survey results

**Figure 7:**
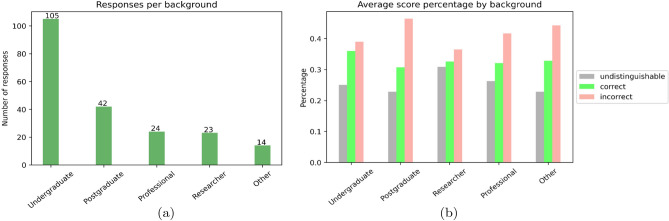
(**a**) Academic or industry experience for individuals with a geoscience related background and (**b**) results of the experiments, with average results by population.

Results of the survey to evaluate the quality of the generated images are presented in Fig. 7. The survey was applied to 205 individuals worldwide from different backgrounds in industry and academia contexts. Most responses come from undergraduate and postgraduate geoscience students (backgrounds are shown in Fig. 7). Although the survey's overall results in various backgrounds were similar, we observe that the performance of participants with different backgrounds (“other” in Fig. 7), is generally lower than those with a geoscience background. Across all background categories, undergraduate students have the highest performance, postgraduates have the lowest performance, and researchers have the highest percentage of doubts. Overall, the survey results show that, on average, the generated images perform better on all backgrounds.

## Discussion

**Figure 8:**
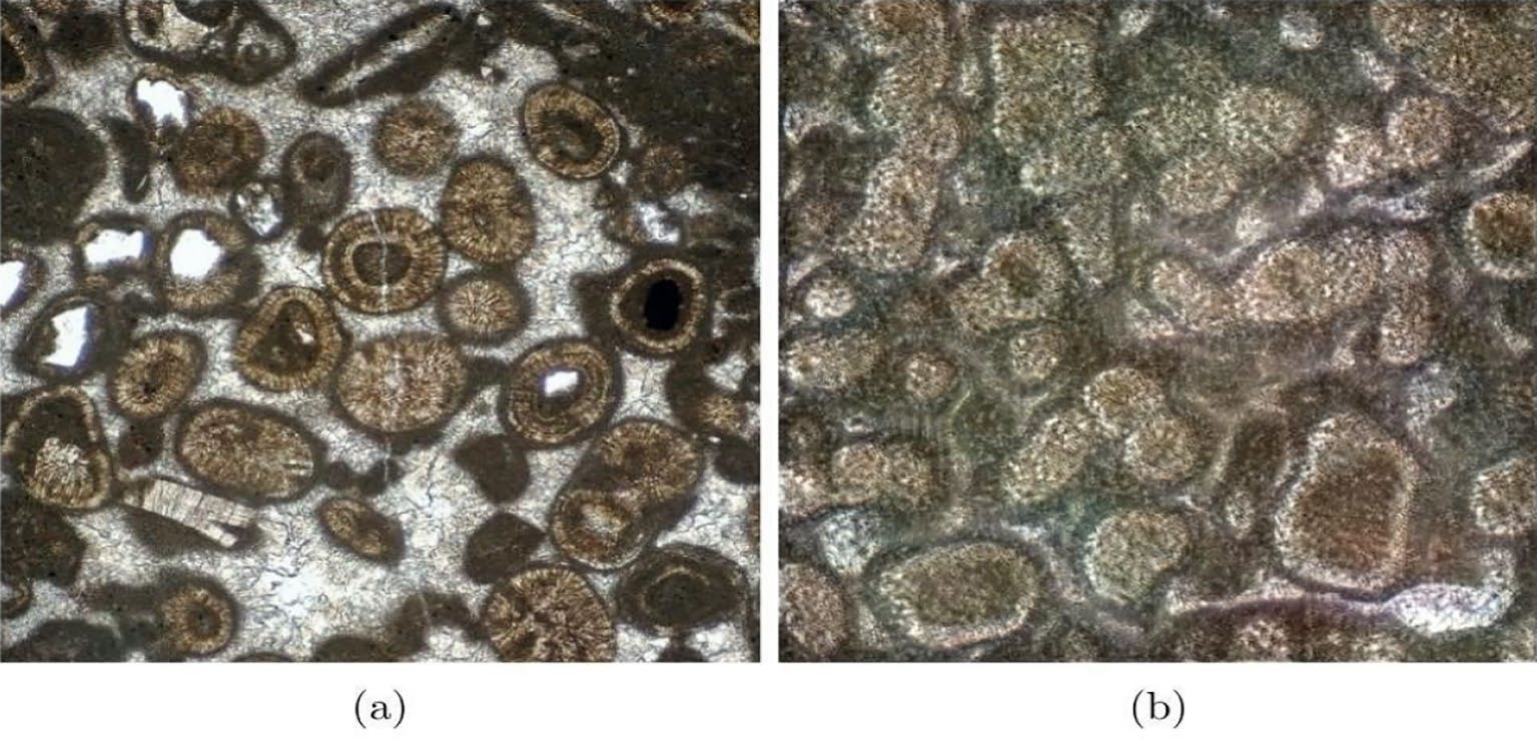
Results of interpolating an image into the model (**a**) Ooilitc limestone^52^, and (**b**) interpolated oolitic limestone in model’s latent space.

**Figure 9:**
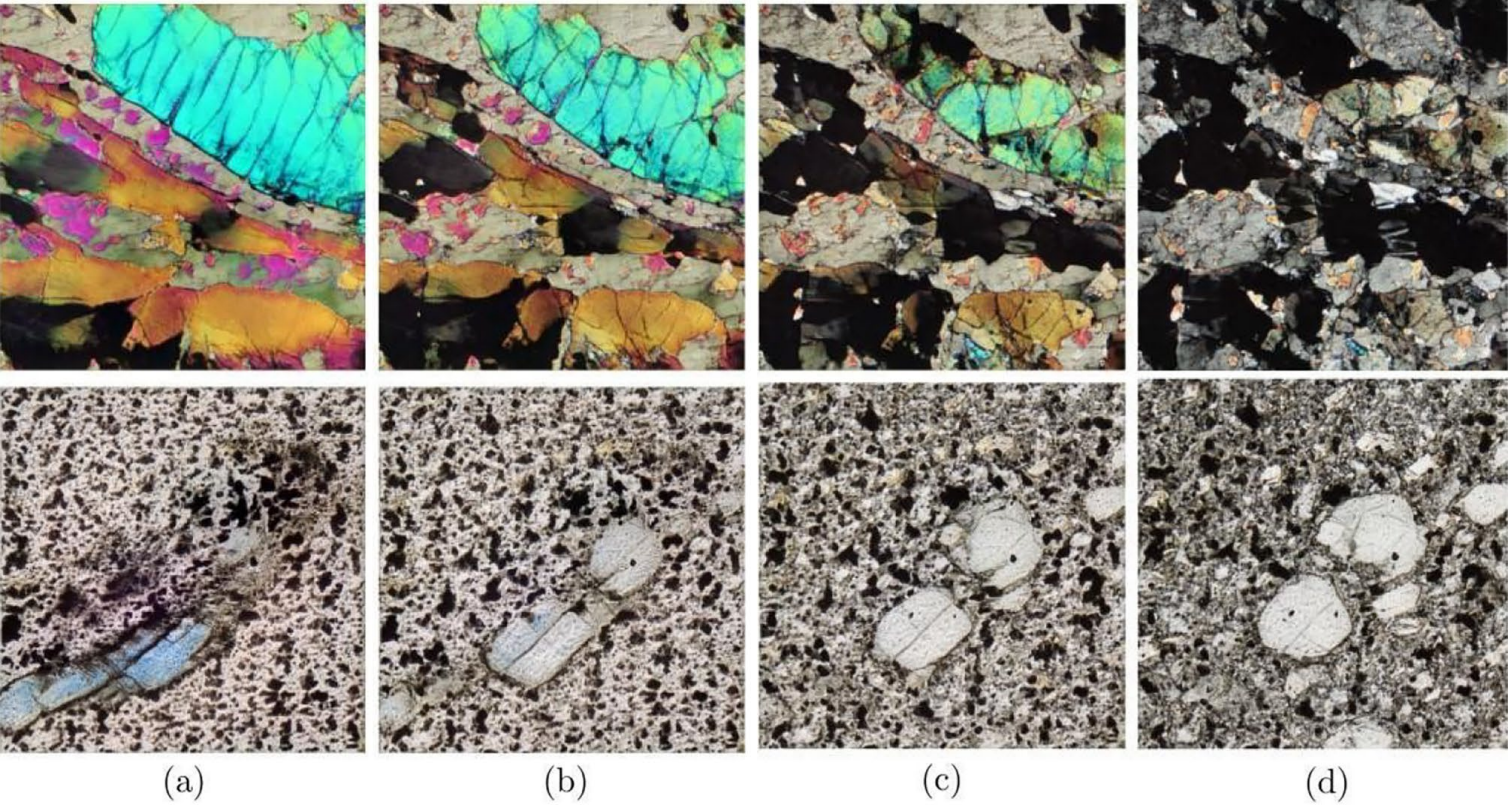
Results of applying truncation to two generated images show a progressive change in truncation and its effects, with (**a**) 1.25, (**b**) 1.0, (**c**) 0.75, (**d**) 0.5 as truncation values.

The proposed use of GAN trained on geological data and with petrographic images enables the visualization of thin sections as a moving system. This could be a way to picture the changing state of different lithologies. Thus far, this application aims to provide a real thin section not seen by the model during training and searching for the associated latent vector. This could lead to similar images found in the model. For example, an oolitic limestone taken from the University of Oxford Rocks Under the Microscope project^52^ is transferred to the model (Fig. 8a), which then proceeds to search for the most similar image within its latent space (Fig. 8b)—resulting in a vector generated for the artificial image found within latent space. The proposed use of this feature is to search for similar thin sections and experiment with proximal vectors and lithology visually. Searching for a similar thin section in latent space could help us visualize how a computer machine learning model organizes a petrographic set of images, which sections it tends to group, and which ones uses to group lithologies in latent space, what features are more dominant, and how to control the most important ones from a geological point of view, e.g., grain size or foliation, to generate specific textures. The Truncation Trick is a modification of the latent distribution by applying a truncation of the normal distribution used to generate images, i.e., truncating the values which fall above a certain threshold^23^. This has been shown to improve and boost the FID score of the generated images and was used in the survey to increase the probability of an artificial thin section appearing as a real one, using 0.7 as a truncation value. The truncation value experimentation generates more unrealistic minerals the greater the threshold value, producing images with varying threshold values as shown in Fig. 9. Conversely, reducing the truncation value produces more down-to-earth minerals, albeit with a tendency to make the general color of the thin section gray.

**Figure 10:**
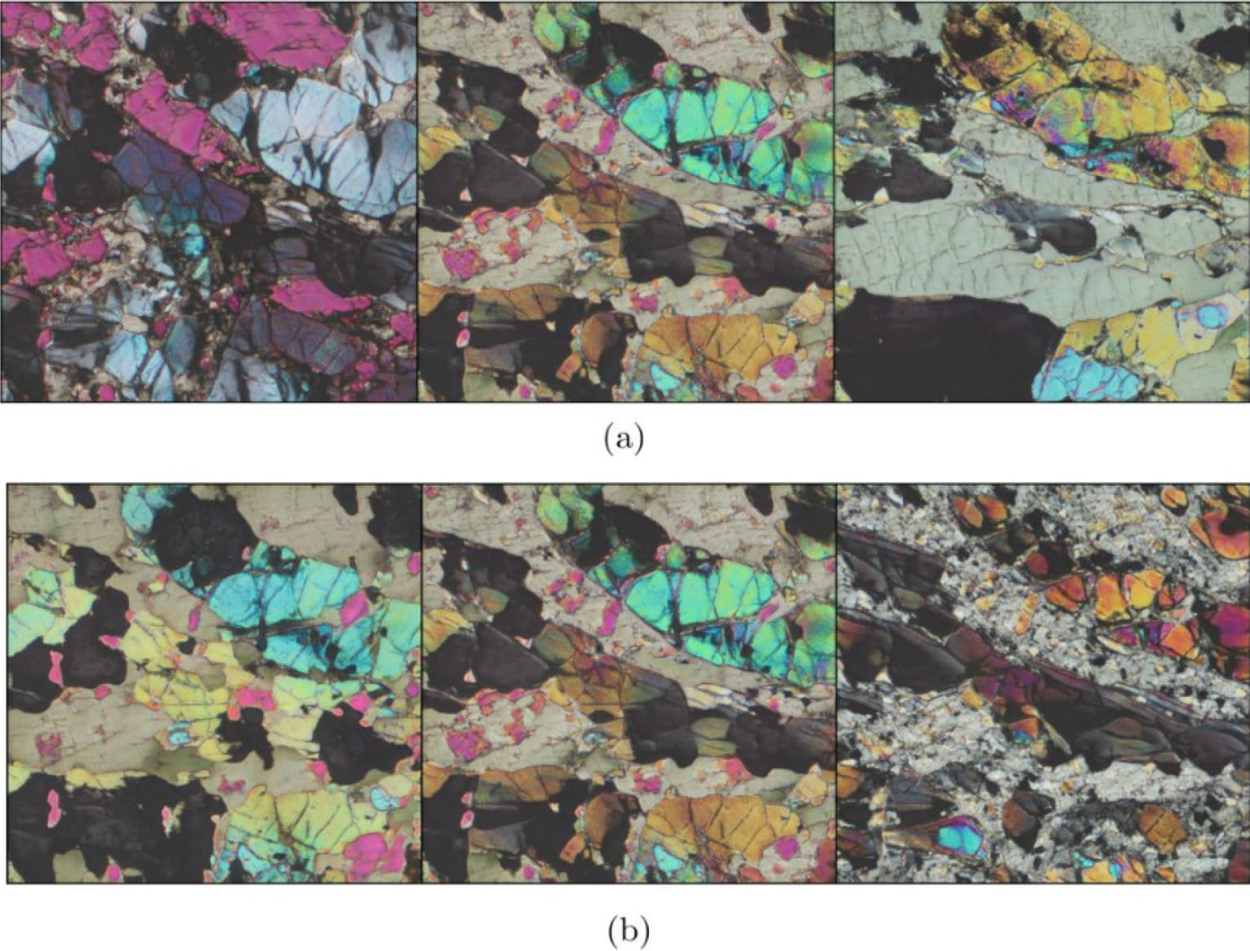
Feature Extraction of important vectors in the model, the same seed, 168,947, in the center is changed (**a**) modifying colors of the minerals and (**b**) changing the percentage of matrix in the thin section.

An application of synthetic data generation is the ability to extract human-readable feature vectors in latent space. We used the Closed-Form Factorization^24^ of latent vectors for the all lithologies 512 × 512 model. This method could be used in the future for visualizing different features being modified on the same mineral assemblage, Fig. 10. Moreover, we could use the trained model to extract vectors that can be used to modify the same thin section and add or remove certain constituents. Future applications of this factorization could be petrographic and petrological modeling, especially if this vector can be associated with certain characteristics of geological environments. An interesting application is grain size modification and the kind of minerals present; this model could also be used to visualize facies and lithological changes and assist in geological workflows that rely heavily on petrographic information.

We also applied this method to an image classification problem, using 200 images of landscapes and 200 artificial thin sections. We trained a deep convolutional neural network architecture and tested the model using images of landscapes and actual thin sections. The model reached 95% accuracy with the training data and 80% with the testing set. Synthetic images were more prone to be classified as real, Fig. 7, than actual thin sections. This phenomenon could be explained because generated images tend to look more like an average thin section, given that they are trained to assimilate an entire distribution of images. This "archetypical" thin section is erroneously classified as the real one compared with a single real thin section in a binary classification task, i.e., real, or fake, when a human is used as the classifier. Images that are 'more real than real' have already been observed in GANs trained with faces10,11, and Gestalt theory has been previously used in deep learning in preprocessing steps to obtain efficient image descriptors for CNN^53^ training. We propose that this "gestalt," i.e., the laws on our ability to make meaningful perceptions of the world^54^, GAN phenomenon could extend to nonfacial geological datasets and that should be considered and further studied. This phenomenon could indicate continuity, memory, similarity, closure, and superior figure in the sense of Gestalt theory regarding our understanding and perception of synthetic and real petrographic data. We attempted to address one of these Gestalt principles with a symmetry test between the real and fake images used for the survey, which were found to have higher symmetry.

**Figure 11:**
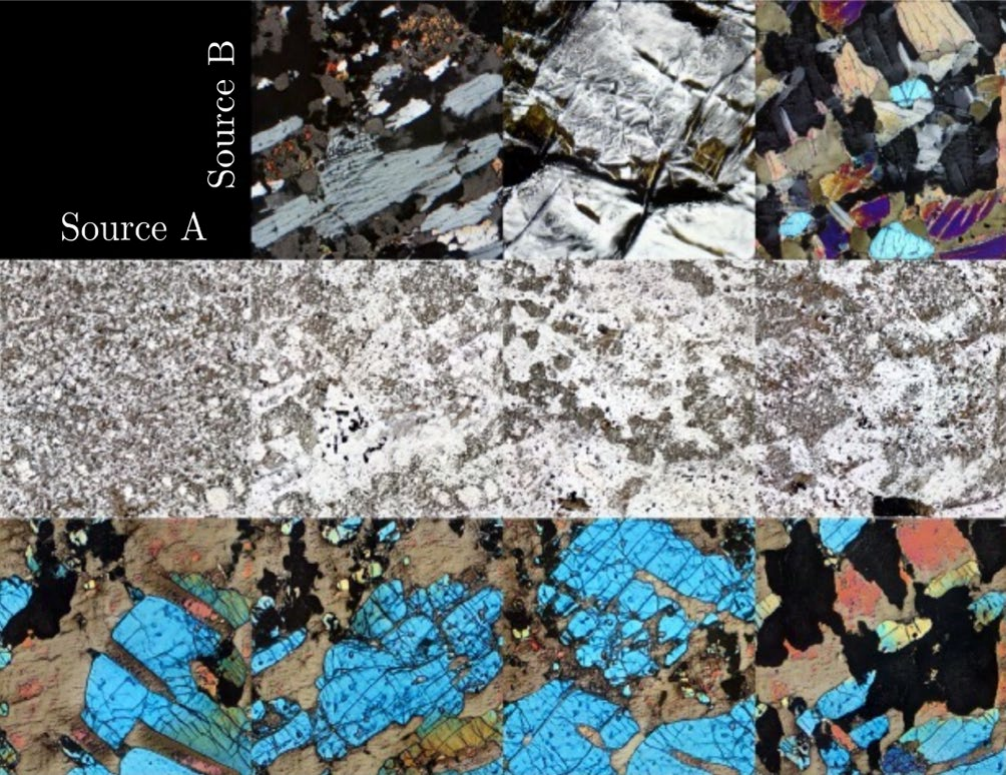
Style-mixing of thin sections from a sample (Source A) to another (Source B).

**Figure 12:**
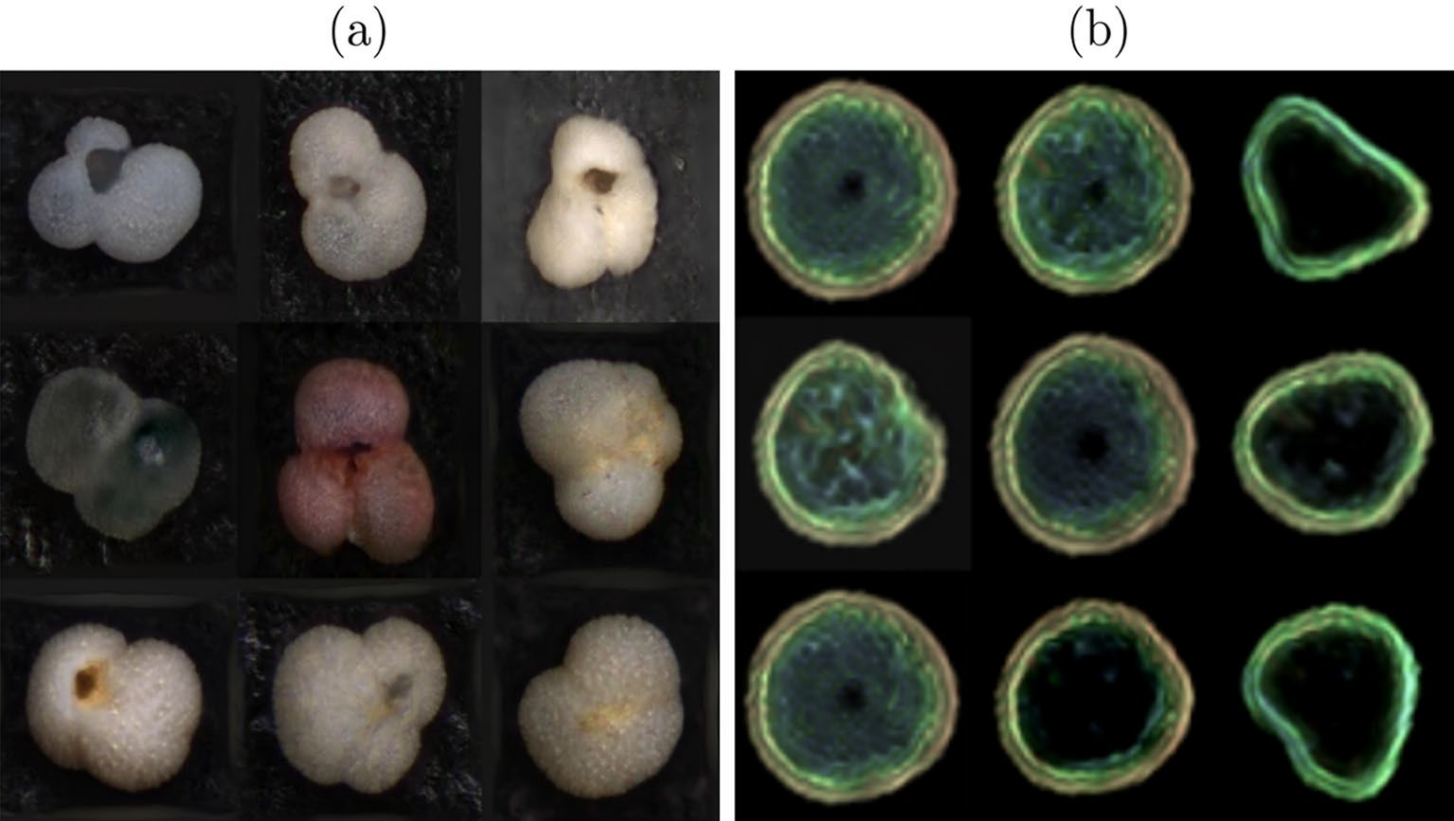
Transferability of the technique to other geological datasets (**a**) Foraminifera Species, (**b**) Pollen Genera.

The significance of this model is enabling the generation of artificial thin sections. With further studies, it could be used as a viable method for dataset augmentation, with the potential as a tool for self-labeling being input to semi-supervised and unsupervised learning algorithms; explainability of this kind of model is also an area of research and could elucidate in the future how a GAN organizes data in its latent space. It is also noted and encouraged that the final model can be used as the starting point for training more domain-specific petrographic datasets, and this could be done through style-mixing of the GAN model, to generate more specific generative models, e.g., in the generation of carbonate constituents^4^ or an only metamorphic thin section generator. The use of style-mixing in a petrographical dataset is shown in Fig. 11, where the model learns parameters such as grain size and XPL/PPL, changing them as the style of the thin section is mixed between domains (Source A and B in Fig. 11). Style-mixing features of interest in a geological dataset could be used to increase diversity or even fill under-represented classes^32^. This architecture also makes it possible for the images to be generated according to a signal, application of this being the audio-reactive GAN "MAUA" implementation^55^. Further exploration and evaluation of the generated thin sections in latent space could aid in evaluating how a given lithological feature evolves. In the future, this could be used to assist in interactive explanation and visualization or modeling of petrographic environments, e.g., the impact of varying levels of metamorphism on a thin section and the effects of change in energy levels in a sedimentary environment. We also observe that, with the different image sizes tested, we expect to get lower scores, i.e., better, for smaller image sizes. A comparison in Table 5 gives us an idea of the dataset size needed to achieve a target FID score. For validation against other geological datasets, we test the same architecture with two other geoscience-related datasets, a foraminifera species-level dataset collected, and a general-level pollen dataset published. Both datasets were collected for CNN-Classification tasks, and we selected the nine foraminifera species and five pollen genera with the most images, i.e., 103. These datasets were resized to 18,166 256^2^ px images for the foraminifera and 7925 64^2^ px images for the pollen dataset, reaching 15.8 and 18.68 FID scores, respectively Fig. 12.

### Future recommendations

We encourage implementing the recently released StyleGAN3 model and upcoming GAN architectures to improve the current model further and use the trained model in more domain-specific datasets. Exploration of latent space and feature modification of thin sections is needed as ways to prove that this type of architecture will help in the visualization of changing variables in geological environments by way of changes in latent space, image-to-image translation is suggested to generate petrographic images from another type of images, and implementation of super-resolution^29^ would be most needed to upsample available petrographic datasets resolutions. Exploration of features extracted from the model is a way forward to control specific geological characteristics of the generated data, i.e., a feature for controlling the grain size, the predominance of the matrix over grains, or the abundance of a particular mineral species. It is also recommended to explore ways to associate latent vectors with geochemical data to visualize the effects of changing modal composition on a thin section; this could be useful, for example, to generate thin sections based on modal composition in metamorphic petrology modeling. A more discrete survey is advised, i.e., generating a model trained on a specific lithology, thus enabling more domain-specific tests to be made, e.g., assessing sedimentologists or petrologists to give an artificial thin section tentative metamorphic or sedimentary facies^56,57^. We tested the GAN model capacity as a tool to generate datasets for other machine learning algorithms. For this, we trained a binary image classifier using a convolutional neural network over 100 synthetic thin sections versus 100 landscape images, the model achieved over 90% accuracy on training and testing, and when tested against 40 real thin sections, the accuracy dropped but was over 80% nonetheless, further validation is needed to use this kind of model as a data augmentation tool in future geoscience workflows.

## Conclusions

1. It is possible to generate an artificial dataset of petrographic thin sections using Generative adversarial networks, via the architecture of StyleGAN2. Training of a viable GAN using StyleGAN2 in this context needs at least 5000 images to achieve sufficiently good images, and more than 10,000 images are recommended to generate an optimal model (i.e., lower than 15 FID score).
2. Based on the result of the survey, we conclude that artificially generated thin sections can be indistinguishable from real ones and even be seen as more authentic than real ones, allowing this tool to generate thin sections of sufficient quality to be able to deceive domain subject experts.
3. Latent space exploration of the model is a method of visualization and interpolation of real thin sections into the model. Further exploration of styles in the context of petrography is needed to support GAN models as a technique for petrographic modeling.
4. Closed form factorization of latent space in a petrographic image generator is used for extracting at least two human readable vectors that could be used in the future for modeling purposes in the geosciences.
5. Both dataset size requirements 10^3^—10^4^ and GPU computing costs prevent the application of GAN-based frameworks, especially in certain geological subfields where data is limited and/or high dimensional.

## Data Availability

The dataset and code used and/or analysed during the current study available from the corresponding author on reasonable request. This is a manuscript under review process and the trained models will be available soon. For the StyleGAN2 + ADA implementation please refer to https://github.com/NVlabs/stylegan2-ada-pytorch.
